# Psychoneuroendocrine Associations with Momentary Pelvic Pain in Endometriosis

**DOI:** 10.1007/s12529-025-10402-w

**Published:** 2025-11-04

**Authors:** Katharina van Stein, Martin Stoffel, Anne Marshall, Ariane Germeyer, Sabine Herpertz, Valery Grinevich, Monika Eckstein, Ditzen Beate

**Affiliations:** 1 Faculty of of Behavioural and Cultural Studies, Ruprecht Karls-University Heidelberg and Heidelberg University Hospital, Heidelberg, Germany; 2 Medical Psychology, Health and Medical University Erfurt, Erfurt, Germany; 3 German Center for Mental Health (DZPG), partner site Heidelberg/Mannheim/Ulm, Germany; 4 Department of Gynaecological Endocrinology and Fertility Disorders, University Women’s Hospital, Heidelberg, Germany; 5 Department of General Psychiatry, Heidelberg University Hospital, Heidelberg, Germany; 6 Department of Neuropeptide Research in Psychiatry, Central Institute of Mental Health, Medical Faculty Mannheim, University of Heidelberg, Mannheim, Germany

**Keywords:** Endometriosis, Psychoneuroendocrinology, Stress, Pain, Social support, Experience sampling

## Abstract

**Background:**

Endometriosis is a gynecological condition which often causes chronic or recurrent pelvic pain (CPP). The disease can thereby impose a significant burden on affected individuals and their romantic relationships. Existing research highlights the substantial influence of stress, social support, and hormonal factors on pain experience, but data from daily life is scarce.

**Methods:**

This ecological momentary assessment (EMA) study aimed to explore the association of stress, partners’ social support styles, cortisol, and oxytocin in daily life with pain experiences among women with CPP (*N* = 66) across 7 days, resulting in a dataset with up to 2100 data points per variable across multiple measures.

**Results:**

Stress was positively correlated with pain ratings both within and between individuals, while no significant associations were observed between salivary cortisol or oxytocin levels and pain ratings. Distracting as well as solicitous social support was positively related to higher pain ratings on a between-person level but showed no or slightly negative associations with pain on a within-person level.

**Conclusion:**

These findings suggest that both stress and social support can adversely impact pain experience in endometriosis. This knowledge is essential for developing comprehensive interventions: While stress management can be beneficial, the role of social support is more intricate, requiring tailored guidance for close others and their support behavior.

**Supplementary Information:**

The online version contains supplementary material available at 10.1007/s12529-025-10402-w.

## Introduction

Endometriosis (EM) is a gynecological disorder defined by the presence of endometrial-like tissue outside the uterus, primarily in the abdominal cavity, pelvic cavity, and ovaries [1]. The disease unfolds depending on the menstrual cycle and can be asymptomatic, but is often associated with dysmenorrhea (pain before and during menstruation), dyspareunia (pain during sexual intercourse), and infertility. Chronic pelvic pain (CPP), in particular, affects up to 80% of patients with EM [2, 3] and substantially impairs quality of life [4–6]. As pain in EM does not correlate with the severity of the disease and might even persist after successful treatment [7, 8], it is crucial to further elucidate pain processes and influential factors ranging from a psychosocial to a molecular level.

From a psychosocial perspective, stress has been associated with pain. In healthy samples and chronic pain patients, studies found that stress renders individuals more susceptible to pain stimuli [9]. In line with this, studies investigating everyday life stress suggest that stress significantly predicts momentary pain levels in women [10]. However, there is some evidence for sex-specific differences in stress and pain processes (for reviews, see [11, 12]), requiring more research on stress and pain-modulating mechanisms in women.

One interpersonal factor that has been extensively investigated in the context of pain—among others in chronic pelvic pain—is social support. However, contrary to the common belief that social support uniformly reduces stress and thereby ameliorates (chronic) pain, previous research reveals a more complex picture [13, 14]: While overall the availability of social support is associated with positive health outcomes [15], the actual receipt of support demonstrates mixed effects, encompassing both positive and, notably, negative outcomes [16, 17]. In a large sample of young women with menstrual pain, perceived social support predicted lower pain-catastrophizing one year later [18]. Close and loving others usually show supportive behavior in response to pain expression, and this might be immediately helpful on a momentary level [19]. However, these initially intuitive and positive interaction patterns might also result in an increased focus on the pain, operant conditioning processes of avoidance behavior, and increasing disability in the long term. This seeming paradox may be solved by focusing on the type of social support. While, to our knowledge, there has not been any research on social support styles in CPP, studies in diverse other chronic pain conditions found specific effects of different types of social support: solicitous support (e.g., taking over tasks and duties) was related to higher pain [20], and distracting support (e.g., encouraging to work on a hobby) to reduced pain [21]. A study by Nees et al. [20] suggested that solicitous support may reinforce pain behavior through operant learning in women suffering from chronic low back pain, thus increasing the risk of secondary gain. If such a couple dynamic evolves, the partner’s mere presence may act as a cue that modulates pain processing [20]. These interaction patterns are particularly salient in the context of chronic pain conditions, which involve sensitive relational issues pertinent to couples, including bodily self-perception, sexuality, family planning, and fertility. Chronic pelvic pain due to endometriosis might therefore be particularly relevant in this context.

On a neuroendocrine level, the hypothalamic-pituitary-adrenal (HPA) axis has been associated with stress and pain perception, and the glucocorticoid hormone cortisol, as one main end-product of HPA axis activation, is frequently interpreted as a biological marker for experienced stress [22–24]. In chronic pain, the HPA axis is often dysregulated, a phenomenon that can impact inflammatory responses [25] and cortisol reactivity [26]. Chronic pain patients have been found to have heightened cortisol responses [27], but, importantly, these data are almost exclusively derived from laboratory studies, while daily life data on cortisol in chronic pain is scarce [10].

The neuropeptide oxytocin also relates to the HPA axis: it dampens physiological and behavioral responses to stress by modulating HPA axis activity [28–30], as well as cardiovascular reactivity [31, 32]. In addition, oxytocin seems to be involved in social functions [33–35] and is proposed to modulate the stress-buffering effect of social support [36, 37]. Oxytocin has also been associated with pain. Exogenous oxytocin administration was linked to reduced pain in healthy samples [38, 39] and in chronic pain patients [40] (however, see contrasting effects in women: [41]). Interestingly, in male patients with chronic lower back pain, intranasally administered oxytocin seems to reduce perceived heat pain, but not spontaneous pain [42]. This suggests that oxytocin’s effects on non-experimental (i.e., naturally occurring) pain may be limited or absent; however, further research is warranted to clarify its role. Furthermore, the few existing studies focusing on endogenous oxytocin levels and pain experience have resulted in mixed findings [43]. But, importantly, low serum oxytocin levels have been linked to dysmenorrhea [44].

Taken together, prior research has begun to elucidate how stress, social support styles, and endogenous cortisol and oxytocin levels relate to pain in CPP, yet important aspects remain insufficiently understood. Based on this, we aimed to investigate the effects of solicitous and distracting support in CPP. To examine both within-person and between-person level effects, as well as associated neuroendocrine mechanisms, an ecological momentary assessment (EMA) study was conceptualized involving CPP patients and their partners. The following hypotheses were pre-registered^1^:

1. Higher subjective stress levels are associated with higher pain levels on the between- and within-person level.
2. Higher distracting social support is associated with lower pain levels on the between- and within-person level.
3. Higher solicitious social support is associated with higher pain levels on the between-person level but lower pain levels on the within-person level.
4. Higher endogenous cortisol levels are associated with higher pain levels on the between- and within-person level.
5. Higher endogenous oxytocin levels are associated with lower pain levels on the between- and within-person level.

## Methods

The data presented here are derived from a pre-registered, overarching project that examined the influence of stress and social support on CPP (principal investigator: Beate Ditzen, Heidelberg University Hospital). Data collection occurred between December 2020 and June 2023. The overarching project received approval from the ethics committee of Medical Faculty Heidelberg (S-126/2019). All participants gave written informed consent and received a reimbursement of 120 Euros.

### Recruitment

**Fig. 1:**
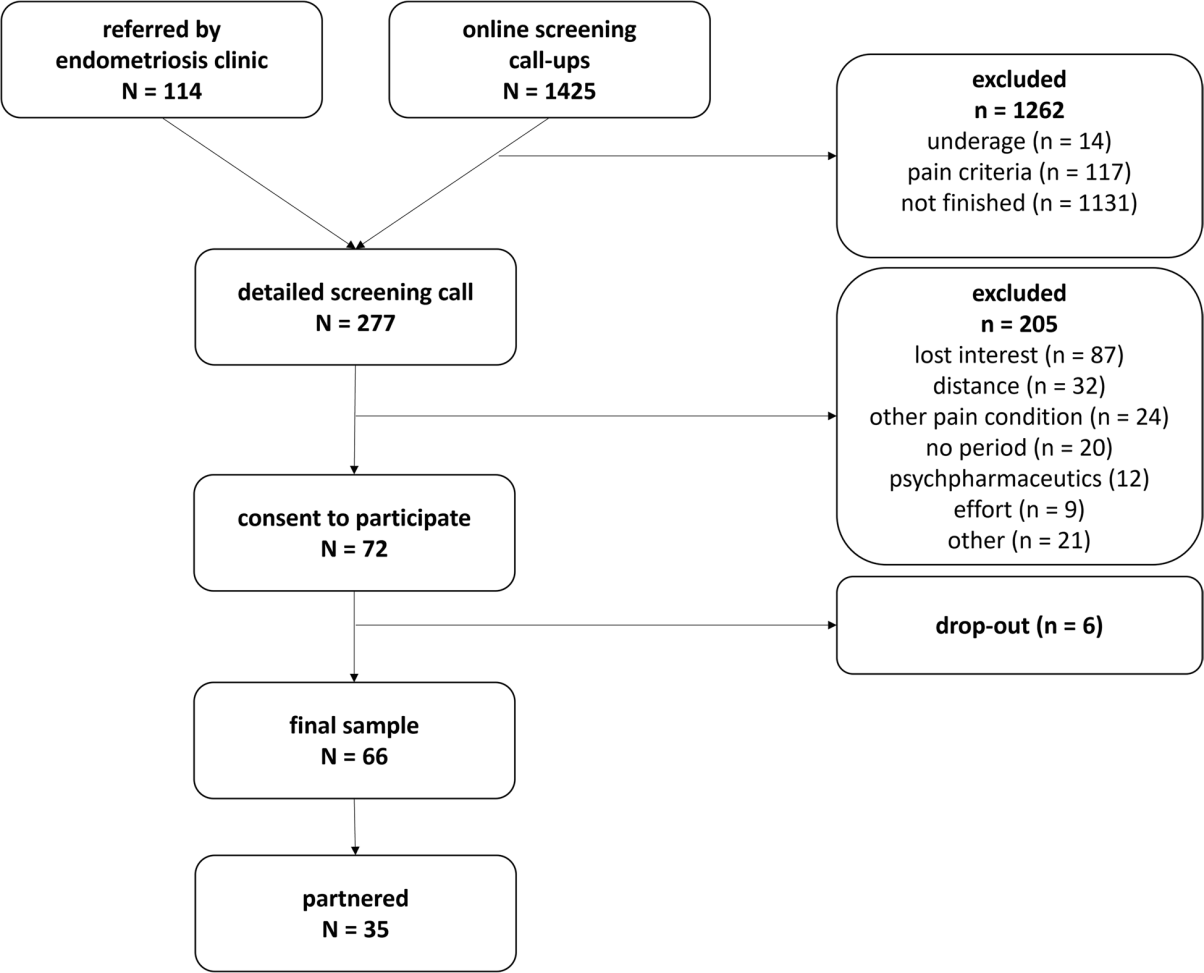
Flowchart of the recruitment process. Participants were recruited between December 2020 and June 2023, via the EM clinic and social media. In total, 72 individuals met the criteria, of whom 66 participants finished the assessment. Out of these, 35 participants were partnered and thus relevant for the social support hypotheses

An initial sample size of *N* = 60 participants^2^ was pre-registered.^3^ Once 60 participants had completed the study, recruitment was discontinued. However, all individuals who had already enrolled were allowed to complete participation, resulting in the final sample size of *N* = 66 women. Mean age was 29.4 (SD = 6.09), ranging from 18 to 43 years. Among the partners, there were 20 men, 1 non-binary person, and 14 individuals who did not specify their gender. The average relationship duration was 6.94 years (ranging from 1 to 17 years). Of the couples, 28 did not have children, 4 had children, and 3 did not provide information. Participants were recruited via the EM Consultation of the Department of Gynecologic Endocrinology and Fertility Disorders at University Women’s Hospital Heidelberg and via social media posts combined with a pain screening (Chronic Pain Grade by Klasen et al., 2004). The main inclusion criterion was chronic or recurrent pelvic pain as reported in the EM Consultation or having a Chronic Pain Grade of 2 or higher. Thus, all participants suffered from CPP, whereas not all had confirmed endometriosis (yet). Also, participants were required to be able to roughly estimate the onset of their next menstrual/withdrawal bleeding. Exclusion criteria were the following: being younger than 18 or older than 45 years, current psychotic disorders, current psychopharmacological treatment, psychoactive medication, preceding alcohol abuse or substance abuse, current hormonal fertility treatment, any kinds of cancer, acute infections, chronic pain disorders apart from CPP, taking the pill in a long-term cycle. EM status was confirmed by laparoscopic surgery by the EM Consultation or by diagnosis letters from other medical institutions. All participants were required to live in a 2-h radius around Heidelberg to ensure the saliva samples remained frozen during the transfer from participants’ homes to the lab. The recruitment process is outlined in the flowchart (Fig. 1).

### Procedure

Participants were pre-screened and informed about the study via phone and received all study documents, a study phone, and saliva collection kits via mail. They filled in a baseline online questionnaire via the platform SoSciSurvey.de before starting the ecological momentary assessment (EMA). Participants were assessed for 7 consecutive days, starting 3 days before the estimated start of the next menstrual/withdrawal bleeding. Thus, data collection was planned to take place during the late luteal and early follicular phases, to cover a high variability in pain intensity.

**Fig. 2:**
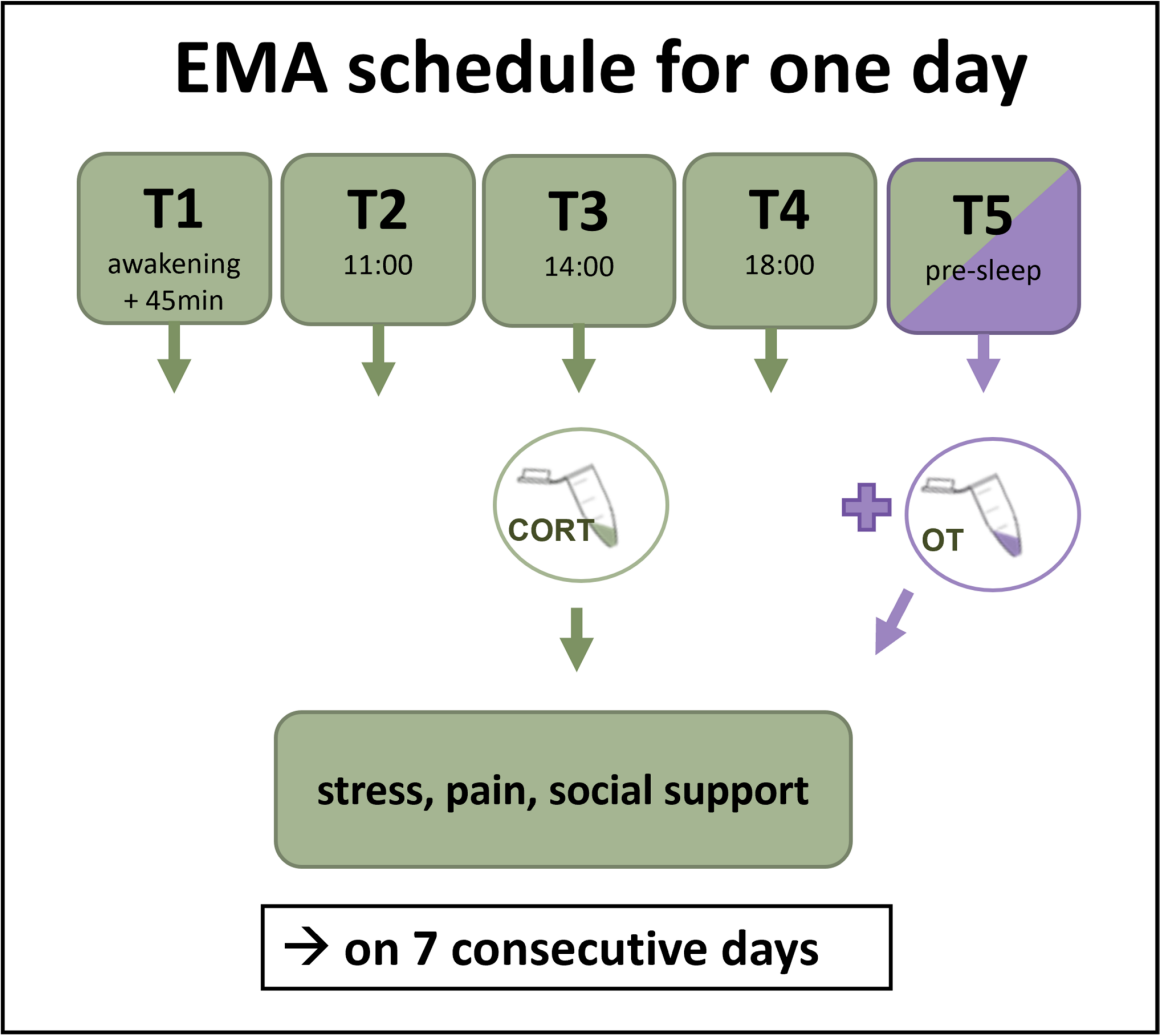
Ecological momentary assessment (EMA) schedule across one day. The figure depicts the time points at which specific variables were assessed during a single study day. At time points T1 to T4, participants provided data on stress, pain, social support, and a saliva sample for cortisol analysis. At T5, assessments additionally included intimacy and a saliva sample for oxytocin analysis. This assessment schedule was repeated over a period of seven consecutive days. Cort = cortisol; OT = oxytocin

Participants were provided with a study phone running the EMA app movisens XS. At 5 times per day, they received prompts to report on social interactions, subjective stress and pain ratings, and to collect saliva samples (see Fig. 2). Except for the awakening and pre-sleep questionnaire, all questionnaires were presented following a time-based schedule. However, prompts were allowed to be postponed to later or to be responded to before the actual measuring time, resulting in a high response rate. Questionnaires were assigned to a measurement time as long as they were not responded to more than 15 min before or after the scheduled measurement time. There were more prompts and additional hormones assessed, which are however not relevant for the aims of this study. For the detailed schedule, see electronic supplement S1.

### Materials

#### Questionnaires

Sociodemographic and basic medical information was assessed with the baseline questionnaire. The EMA questionnaire always started with a directive to collect a saliva sample and accompanying quality control questions. Subsequently, various items regarding the psychosocial situation followed. The processing time averaged around 5 min. For this study, the following items were relevant:

- Pain was assessed with a single item (“Estimate the extent of your current pain (right now)!”) on a 7-point Likert scale ranging from 0 (no pain) to 6 (the most extreme pain I have ever experienced).
- Stress was assessed with a single item (“How nervous and stressed do you feel at the moment?”) on a 7-point Likert scale ranging from 1 (not at all) to 7 (very much).

Information on analgetic use was taken from a general question on medication use since the last prompt.

In the pre-sleep questionnaire, participants were asked if their menstrual/withdrawal bleeding started on this day with a single item.

Social support was assessed in a subsample of participants who were in a relationship and who spent a significant amount of time with their partner.^4^ Here, part II of the German version of the Multidimensional Pain Inventory (MPI; Kerns et al., 1985) assessing social support styles was used. Participants were asked how their partners reacted to their pain, and 11 different partner reactions were suggested. Only reactions coded as solicitous (e.g., ‘gives me a massage’) or distracting (e.g., ‘encourages me to work on a hobby’) are considered in this paper. For each item, participants indicated support on a 7-point Likert scale ranging from 0 (not at all) to 6 (very much). Preceding filtering questions ensured that the partner was present at the time of assessment and that the participant was experiencing pain. As a result, the number of social support events was limited.

#### Neuroendocrine Measures

During 7 days at 5 times per day, participants self-sampled their saliva into plastic collection tubes via passive drool technique and immediately stored each sample in their home fridges (samples for cortisol assessment) or freezers (samples for oxytocin assessment). After participation, the saliva samples were stored at −80 °C for no longer than six months before analysis in the biochemical lab at the Institute of Medical Psychology at Heidelberg University Hospital.

Due to the rapid degradation of oxytocin compared to cortisol, oxytocin saliva samples need to be immediately frozen after collection. To maintain sample quality without significantly disrupting daily life, only pre-sleep saliva samples that were instructed to be put into the freezer immediately were used for oxytocin analysis. As a result, only 7 oxytocin samples per participant are available, compared to *n* = 7 × 5 = 35 cortisol samples.

To analyze salivary oxytocin (sOxy) concentrations, saliva samples were thawed and centrifuged at 4 °C at 1500 × *g* for 15 min and subsequently analyzed without extraction (20% of the samples in duplicates) following the protocol of oxytocin enzyme-linked immunosorbent assay from Enzo Life Sciences (ELISA; ENZO Life Sciences, Switzerland). The detection limit was 15 pg/ml, and the intra- and inter-assay coefficients of variation (CVs) were 8.46 and 11.45%, respectively. 1% of values was too high or low to be analyzed correctly and was thus excluded.

Salivary cortisol (sCort) was measured with an enzyme-linked immunosorbent assay (ELISA; Demeditec, DES6611) with 20% in duplicates and a reported detection limit between 0.1 and 30 ng/ml. The intra- and inter-assay CVs in our sample were 2.85 and 5.78%, respectively.

### Data Analysis

Data were analyzed using R version 4.3.1 [45]. To account for the nested structure of the data, the package “nlme” (version 3.1–163.1) [46] was used to fit multilevel models with a maximum likelihood method of estimation. Missing data were handled using Restricted Maximum Likelihood, which inherently accommodates missing data by using all available data points. Measurements within days (level 1; L1) were treated as nested in days (level 2; L2), which were treated as nested in person on level 3 (L3). To avoid violations of model assumptions (e.g., normality of residuals), distributional properties of all dependent variables were checked prior to fitting the models. In case of non-normality, data were transformed using Box-Cox transformations [47]. As pre-registered, all models included the following control variables: age, contraceptive pill intake, cycle day, analgesic intake, and time within days. All control variables (apart from time within days) were centered on their grand mean. Potentially nonlinear time trends were tested by comparing models with linear time and models with higher-order polynomials. Thereafter, random slopes were tested separately by comparing the baseline model (i.e., all covariates, including time within days, and random intercepts on L2 and L3) against the same model with one additional random slope on L2 or L3 [48]. Model comparisons were conducted using likelihood ratio tests and by interpreting the Bayesian Information Criterion (BIC; where a lower BIC indicates a better fit to the data). Following these steps, the focal predictors were entered into the models. Given that they were measured repeatedly on each day for each person, within- and between-person variances were disentangled [49] by person-mean centering each single measurement (within-person effects) and by grand-mean centering each person-mean (between-person effects). As a result, it was possible to draw conclusions regarding (a) whether overall higher/lower levels of the focal predictor would be associated with overall higher/lower levels of the dependent variable (between persons) and (b) whether within-person fluctuations (i.e., higher or lower levels of a predictor for a person as compared to their usual level of this predictor) would be associated with alterations in the dependent variable (on L1). For each final model, model assumptions were tested according to standard procedures (as in [48, 50]). As a last step, sensitivity analyses were performed for each final model in which all observations for each variable in the model with values exceeding ± 3 SD from each respective grand mean were excluded. Graphical representations of model results were built using the packages effects [51] and ggplot2 [52].

## Results

**Fig. 3:**
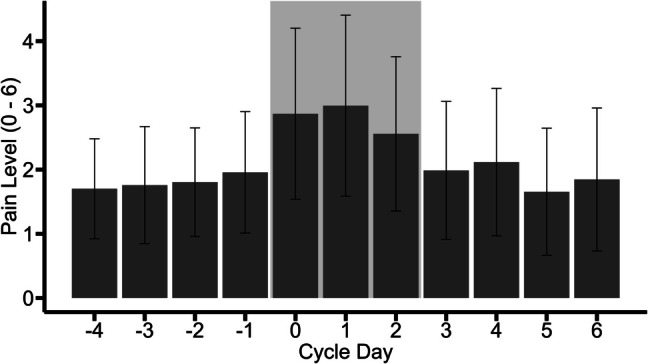
Variation of pain ratings by cycle day. In Cycle Day, zero refers to menstrual onset, negative days signify days before menstrual onset, and positive days signify days after menstrual onset. The shaded area represents the menstrual phase

Of all 66 participants, 35 participants (53%) were in a committed relationship. Seven participants (11%) used hormonal contraception, 11 participants (17%) had up to 10 years of schooling, 27 (41%) had 10–13 years of schooling, 21 (32%) had a university degree, and the educational data of 7 (11%) was missing. In the 7-day window of 66 participants, 45 onsets of menstrual/withdrawal bleeding were reported. Thirty-five participants (53%) had a verified EM diagnosis. Variation of pain ratings by daily measurement time and by cycle day is reported in Table 1, the latter is also depicted in Fig. 3. Descriptive statistics of the outcome and predictor variables are reported in Table 2.

**Table 1:** Variation in pain ratings by time

	*N*	*M*	SD
Daily measurement time			
T1—45 min after awakening	65	1.97	1.21
T2—11:00	404	2.07	1.16
T3—14:00	409	2.12	1.23
T4—18:00	382	2.15	1.17
T5—pre-sleep	403	2.18	1.19
Cycle day			
−4	68	1.70	0.78
−3	106	1.76	0.91
−2	146	1.80	0.85
−1	191	1.96	0.95
0	226	2.87	1.33
1	215	3.00	1.41
2	215	2.56	1.20
3	148	1.99	1.08
4	106	2.12	1.15
5	78	1.65	0.99
6	39	1.85	1.11

Notes: T5 was self-initiated, for T2–T4 data was included with a tolerance of ± 15 min. The low *N* of T1 is partly due to technical difficulties, partly due to participants not having the time to respond to all prompts in the morning (see full EMA schedule in supplement Figure S1). In *Cycle day*, zero refers to menstrual onset

**Table 2:** Descriptive statistics of outcome and predictor variables

	*N*	*M*	SD	Range
Pain	1985	2.1	1.19	1–7
Pain (transformed)	1985	0.74	0.72	0–2.94
Distracting support	328	2.16	1.42	1–6.33
Solicitous support	328	2.89	1.97	1–7
Stress	2100	2.41	1.48	1–7
sCort	2081	5.2	4.6	0.12–29.74
sOxy	396	116.21	129.07	4.4–1135.4

Notes: *sCort*, salivary cortisol; *sOxy*, salivary oxytocin

There were no severe violations of model assumptions in any of the models reported. Detailed results of each model, including the number of observations and the random effect’s structure, are shown in Tables 3, 4, 5, 6 and 7. The exclusion of outliers in sensitivity analyses did not change the magnitude or direction of any of the results reported.

**Table 3:** Pain as a function of stress

	Fixed effects		
	Estimates (se)		*p*
Intercept	0.815 (0.0749)		< 0.001***
Time	0.013 (0.007)		0.082
Age	0.001 (0.011)		0.932
Contraceptives	−0.055 (0.221)		0.805
Analgesic intake	0.402 (0.060)		< 0.001***
Cycle day	0.018 (0.020)		0.378
Stress			
Between-person level	0.185 (0.065)		0.001**
Within-person level	0.080 (0.013)		< 0.001***
	Random effects (SD)		
Level 3 (across persons)			
Intercept	0.370		
Cycle day	0.101		
Stress (within-person level)	0.026		
Level 2 (across days)			
Intercept	0.508		
Time	0.072		
Stress (within-person level)	0.045		
Residual	0.382		

*Note.* Table depicts point estimates (standard errors for fixed effects in brackets). Stress was centered on the person mean (within-person level) and on the grand mean (between-person level); time was not centered and represents the amount of time (in minutes) since midnight; all other predictors were centered on the grand mean. Covariances between random effects within a level were estimated (unstructured random effect matrices). Number of participants = 41; total number of observations = 1366. *p < 0.05; ** *p* < 0.01; ***p < 0.001

**Table 4:** Pain as a function of distracting support

	Fixed effects		
	Estimates (se)		*p*
Intercept	1.217 (0.112)		< 0.001***
Time	0.00 (0.000)		0.971
Age	−0.013 (0.011)		0.255
Contraceptives	−0.460 (0.362)		0.224
Analgesic intake	0.198 (0.112)		0.080
Cycle day	0.049 (0.023)		0.037*
Distracting support			
Between-person level	0.151 (0.068)		0.043*
Within-person level	−0.034 (0.045)		0.456
	Random effects (SD)		
Level 3 (across persons)			
Intercept	0.202		
Distracting support within-person level	0.005		
Level 2 (across days)			
Intercept	0.296		
Distracting support within-person level	0.125		
Residual	0.332		

*Note.* Table depicts point estimates (standard errors for fixed effects in brackets). Distracting support was centered on the person mean (within-person level) and on the grand mean (between-person level); time was not centered and represents the amount of time (in minutes) since midnight; all other predictors were centered on the grand mean. Covariances between random effects within a level were estimated (unstructured random effect matrices). Number of participants = 18; total number of observations = 184. *p < 0.05; **p < 0.01; ***p < 0.001

**Table 5:** Pain as a function of solicitous support

	Fixed effects		
	Estimates (se)		*p*
Intercept	1.289 (0.109)		< 0.001***
Time	−0.000 (0.000)		0.776
Age	−0.009 (0.011)		0.406
Contraceptives	−0.178 (0.333)		0.600
Analgesic intake	0.159 (0.111)		0.155
Cycle day	0.051 (0.024)		0.033*
Solicitous support			
Between-person level	0.105 (0.045)		0.034*
Within-person level	0.045 (0.044)		0.308
	Random effects (SD)		
Level 3 (across persons)			
Intercept	0.190		
Solicitous support within-person level	0.085		
Level 2 (across days)			
Intercept	0.315		
Solicitous support within-person level	0.002		
Residual	0.328		

*Note.* Table depicts point estimates (standard errors for fixed effects in brackets). Solicitous support was centered on the person mean (within-person level) and on the grand mean (between-person level); time was not centered and represents the amount of time (in minutes) since midnight; all other predictors were centered on the grand mean. Covariances between random effects within a level were estimated (unstructured random effect matrices). Number of participants = 18; total number of observations = 184. *p < 0.05; **p < 0.01; ***p < 0.001

**Table 6:** Pain as a function of cortisol

	Fixed effects		
	Estimates (se)		*p*
Intercept	0.912 (0.084)		< 0.001***
Time	0.002 (0.009)		0.830
Age	−0.000 (0.013)		0.995
Contraceptives	0.089 (0.232)		0.705
Analgesic intake	0.422 (0.061)		< 0.001***
Cycle day	0.023 (0.021)		0.272
Cortisol			
Between-person level	−0.060 (0.048)		0.227
Within-person level	−0.002 (0.004)		0.517
	Random effects (SD)		
Level 3 (across persons)			
Intercept	0.407		
Cycle day	0.107		
Cortisol (within-person level)	0.010		
Level 2 (across days)			
Intercept	0.523		
Time	0.071		
Cortisol (within-person level)	0.000		
Residual	0.392		

*Note.* Table depicts point estimates (standard errors for fixed effects in brackets). Cortisol was centered on the person mean (within-person level) and on the grand mean (between-person level); time was not centered and represents the amount of time (in minutes) since midnight; all other predictors were centered on the grand mean. Covariances between random effects within a level were estimated (unstructured random effect matrices). Number of participants = 41; total number of observations = 1334. *p < 0.05; **p < 0.01; ***p < 0.001

**Table 7:** Pain as a function of oxytocin

	Fixed effects		
	Estimates (se)		*p*
Intercept	0.908 (0.081)		< 0.001***
Time	0.003 (0.008)		0.699
Age	0.013 (0.013)		0.290
Contraceptives	0.179 (0.243)		0.466
Analgesic intake	0.367 (0.062)		< 0.001***
Cycle day	0.016 (0.023)		0.485
Oxytocin			
Between-person level	0.000 (0.001)		0.858
Within-person level	0.000 (0.000)		0.602
	Random effects (SD)		
Level 3 (across persons)			
Intercept	0.396		
Cycle day	0.111		
Oxytocin (within-person level)	0.001		
Level 2 (across days)			
Intercept	0.538		
Time	0.000		
Residual	0.382		

*Note.* Table depicts point estimates (standard errors for fixed effects in brackets). Oxytocin was centered on the person mean (within-person level) and on the grand mean (between-person level); time was not centered and represents the amount of time (in minutes) since midnight; all other predictors were centered on the grand mean. Covariances between random effects within a level were estimated (unstructured random effect matrices). Number of participants = 39; total number of observations = 1247. *p < 0.05; **p < 0.01; ***p < 0.001

### Model 1. Pain as a Function of Perceived Stress

**Fig. 4:**
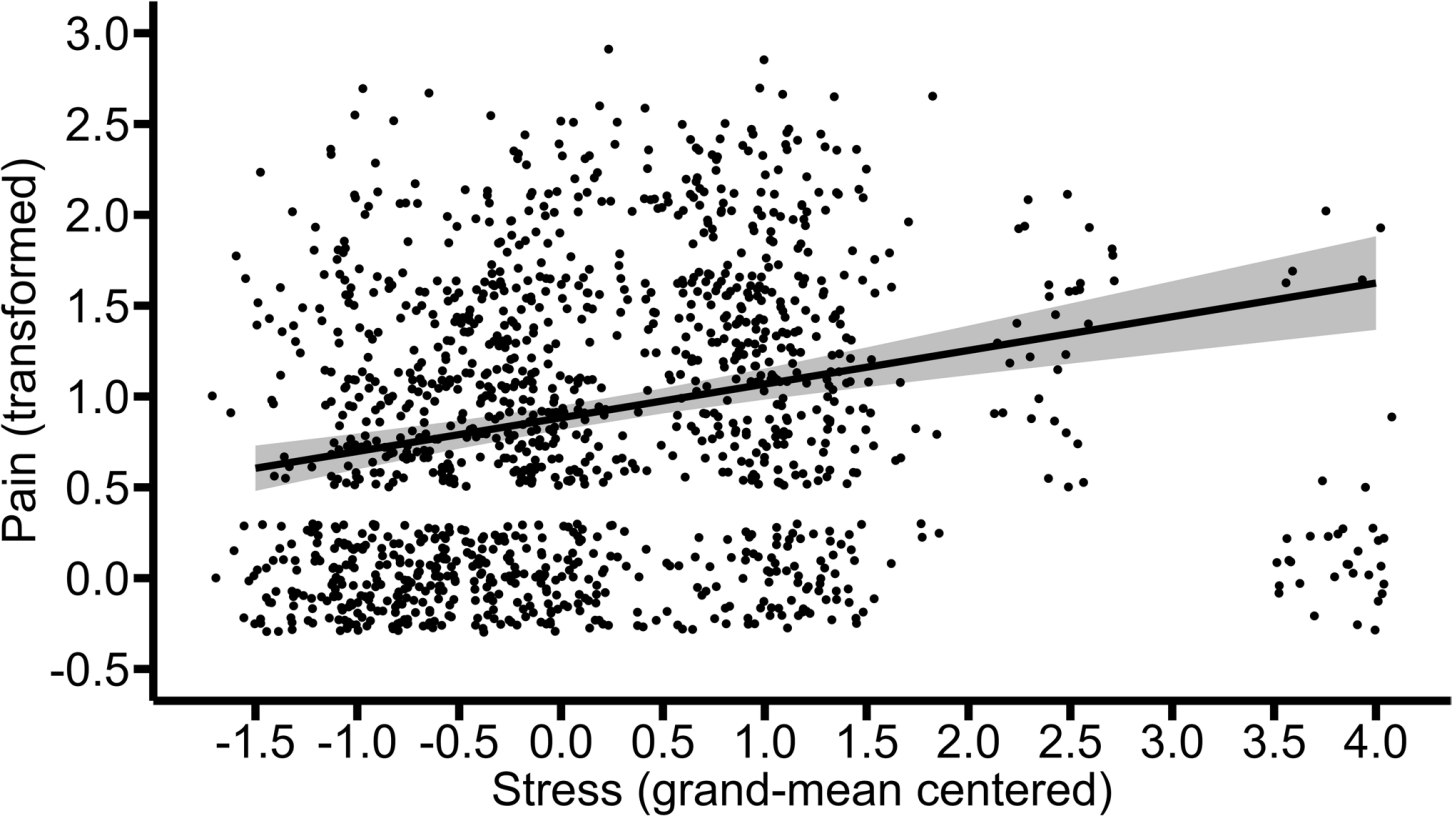
Between-person association of stress with pain. The graph illustrates the average predicted values of pain (transformed) as a function of stress. The ribbon indicates the standard error for the fixed effect. To avoid overlapping data points, they were jittered (adding small random noise) along the x- and y-axis

**Fig. 5:**
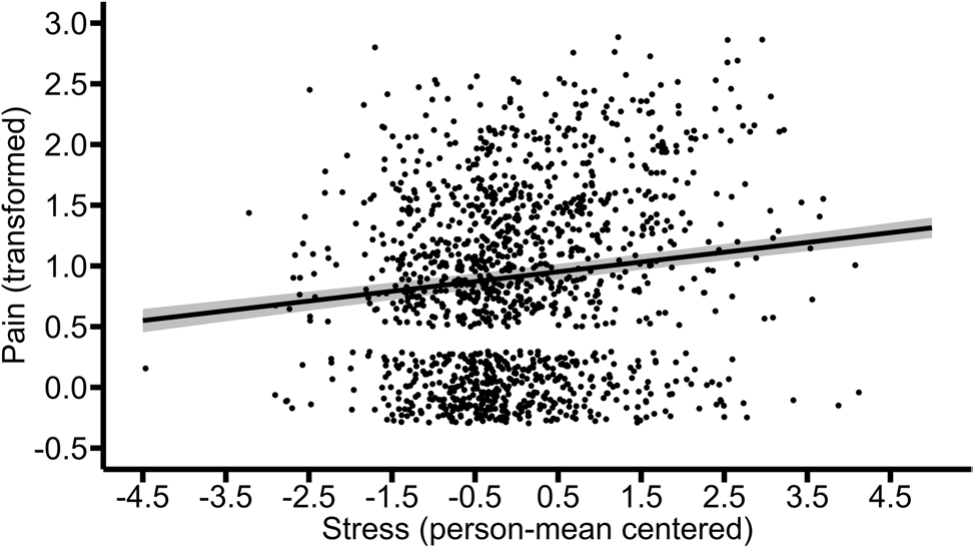
Within-person association of stress with pain. The graph illustrates the average predicted values of pain (transformed) as a function of stress. The ribbon indicates the standard error for the fixed effect. To avoid overlapping data points, they were jittered (adding small random noise) along the x- and y-axis

A continuous autoregressive correlation structure of order 1 (CAR-structure) based on time between assessment (3-h intervals) was added to the final model, because the residual values of L1 were not distributed independently. The updated model (BIC = 2107.84) significantly improved the model fit (*Χ*^2^(2) = 17.57, *p* < 0.001), as compared to the model without the CAR-structure (BIC = 2118.19). Thus, the results reported here are based on the model including the CAR-structure. The within- and between-person variations in stress were significantly and positively associated with pain (between-person: *b* = 0.19, *p* < 0.001; within-person: *b* = 0.08, *p* = 0.001). This indicates that individuals who on average experienced higher stress reported higher average levels of pain. On the other hand, higher levels of stress for a given person (in a given moment within days, as compared to the person’s average levels of stress) were associated with higher levels of momentary pain. Figures 4 and 5 illustrate these effects.

### Model 2. Pain as a Function of Distracting Support

**Fig. 6:**
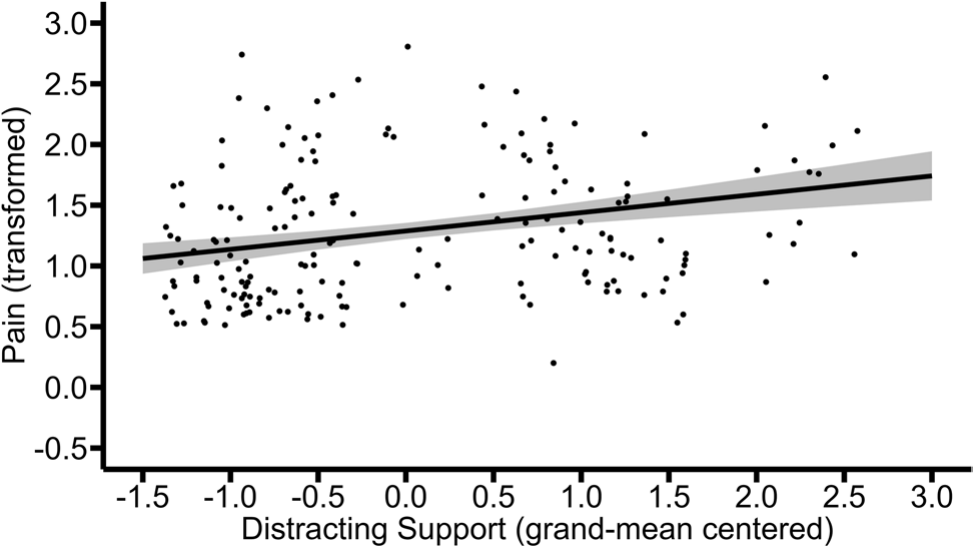
Between-person association of distracting support with pain. The graph illustrates the average predicted values of pain (transformed) as a function of distracting support. The ribbon indicates the standard error for the fixed effect. To avoid overlapping data points, they were jittered (adding small random noise) along the x- and y-axis

**Fig. 7:**
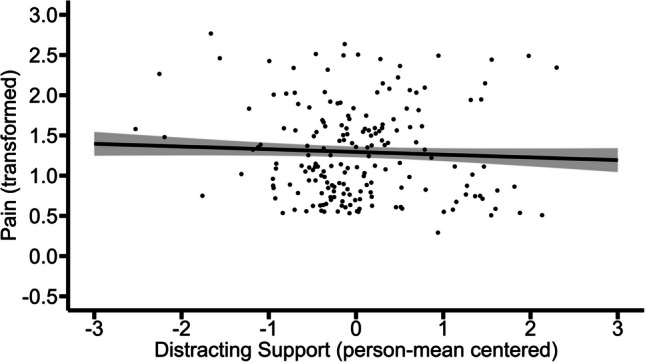
Within-person association of distracting support with pain (non-significant). The graph illustrates the average predicted values of pain (transformed) as a function of distracting support. The ribbon indicates the standard error for the fixed effect. To avoid overlapping data points, they were jittered (adding small random noise) along the x- and y-axis

Only data to which the following applies was used for this model: (a) the participant was in a relationship; (b) in that specific situation the partner was present; and (c) in that specific situation the participant was in pain. The results of this model showed that between-person variations (*b* = 0.15, *p* = 0.04), but not within-person variations (*b* = −0.03, *p* = 0.46), in distracting support were positively associated with pain. This suggests that individuals who on average experienced higher distracting support than others reported higher average levels of pain. In contrast, within these individuals, moments of distracting support receipt were minimally and non-significantly associated with lower levels of momentary pain. Figures 6 and 7 illustrate these effects.

### Model 3. Pain as a Function of Solicitous Support

**Fig. 8:**
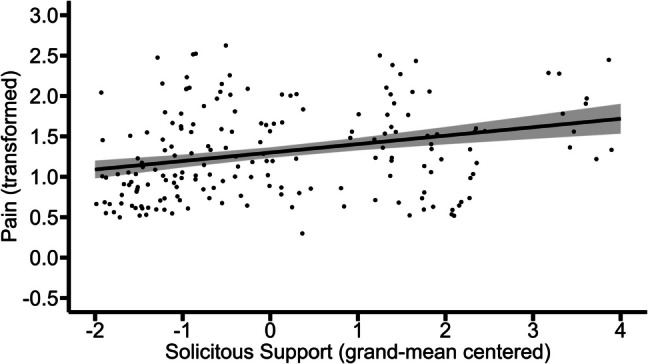
Between-person association of solicitous support with pain. The graph illustrates the average predicted values of pain (transformed) as a function of solicitous support. The ribbon indicates the standard error for the fixed effect. To avoid overlapping data points, they were jittered (adding small random noise) along the x- and y-axis

**Fig. 9:**
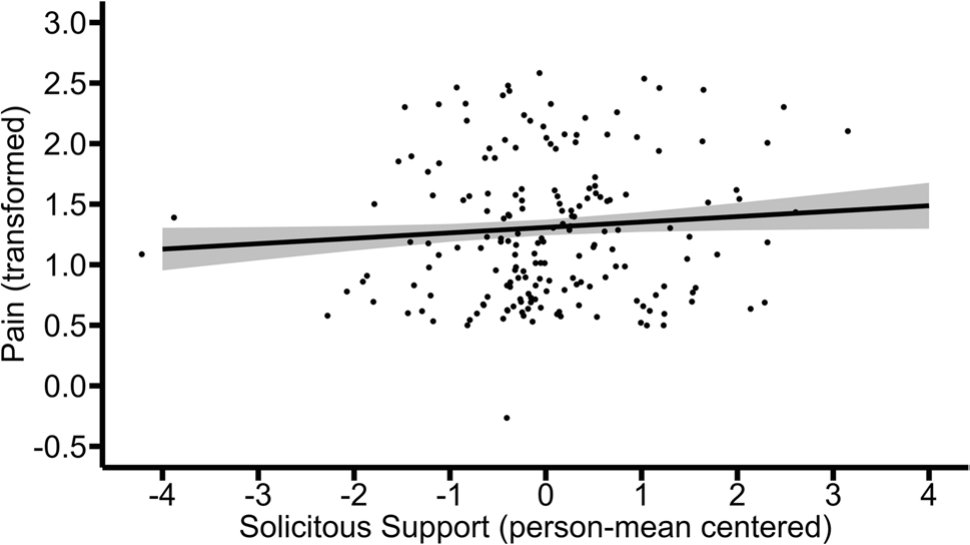
Within-person association of solicitous support with pain (non-significant). The graph illustrates the average predicted values of pain (transformed) as a function of solicitous support. The ribbon indicates the standard error for the fixed effect. To avoid overlapping data points, they were jittered (adding small random noise) along the x- and y-axis

Only data to which the following applies was used for this model: (a) the participant was in a relationship; (b) in that specific situation the partner was present; and (c) in that specific situation the participant was in pain. The results of this model showed that between-person variations (*b* = 0.10, *p* = 0.03), but not within-person variations (*b* = 0.04, *p* = 0.31), in solicitous support were positively associated with pain. This indicates that those who on average experienced higher solicitous support reported higher average levels of pain. Within these individuals, moments of solicitous support receipt were not associated with momentary pain levels. Figures 8 and 9 illustrate these effects.

### Models 4 and 5. Pain as a Function of Cortisol and Oxytocin

A CAR-structure based on time between assessment (3-h intervals) was added to the final models predicting sCort and sOxy, because the residuals on L1 were not distributed independently. The updated model (sCort BIC = 2117.81; sOxy BIC = 1934.70) significantly improved the model fits (sCort *Χ*^2^(2) = 17.74, *p* < 0.001; sOxy *Χ*^2^(2) = 10.26, *p* = 0.001), as compared to the models without the CAR-structures (sCort BIC = 2128.35; sOxy BIC = 1937.82). Thus, the final results reported here are based on the models including the CAR-structures. There were neither within- nor between-person associations of sCort (5 daily measurements, between-person: *b* = −0.06, *p* = 0.23; within-person: *b* < −0.01, *p* = 0.52) or sOxy (1 daily measurement, between-person: *b* < 0.01, *p* = 0.86; within-person: *b* < 0.01, *p* = 0.60) with pain. Graphical representations of these effects are shown in the electronic supplementary information (Figures S2, S3, S4, S5).

## Discussion

The present study investigated the effects of stress, different types of social support, on momentary pain ratings and the association with salivary hormones in women suffering from CPP. Study participants were assessed during a time when pain variability was highest: during the late premenstrual and early follicular phases of their menstrual cycle.

Reported stress was found to be related to higher pain ratings on a within- and between-person level. The data suggested that overall, both distracting as well as solicitous support were related to *higher* pain ratings on a between-person level. No relation emerged for cortisol or oxytocin levels with pain.

### Stress

Matching our hypotheses, we found higher stress ratings to be related to higher pain ratings. This is in line with laboratory studies [9] as well as ecological momentary assessments [10]. Although stress and pain are similar constructs in psychophysiological terms, the medium size *B* coefficients suggest that conceptually different constructs were measured. The present data support the notion that stress and pain are strongly intertwined and can result in a vicious circle: Stress can influence pain signaling, possibly leading to increased pain experience, while pain may evoke feelings of threat, which in turn facilitate HPA activity, increase alertness and attention to potential threat, and facilitate threat learning, all resulting in the experience of stress [53].

### Social Support

On a between-person level, distracting support was associated with higher pain levels. In contrast, on a within-person level, distracting support was slightly (and non-significantly) associated with decreased pain levels. This shift from a positive association of distracting support and pain on the between-person level to a negative association on a within-person level suggests complex interpersonal dynamics and there are different possibilities to interpret this finding: Firstly, on the individual level, self-distraction alone does not seem to be an effective technique to lower pain intensity or pain distress over time [54]. Secondly, on the interpersonal level, distracting support may be weakly, but negatively correlated with patients’ pain acceptance [55]. Thirdly, while on a momentary level, distraction can immediately alleviate pain symptoms, social support overall might increase focus on the symptoms in the long-term.

For solicitous support, results imply that this support style may be pain increasing on both the between- and within-person levels—although the within-person effect was not significant and should thus be interpreted cautiously. This interpretation is in line with recent findings by Nees et al. [20] showing that women suffering from chronic low back pain had stronger pain-related neural responses and higher pain ratings when their partner with a solicitous support style was present. The results suggest that operant learning mechanisms are involved and that a partner with a solicitous support style might unintentionally serve as a cue to process pain stimuli differently. Above this, solicitous support may decrease the patients’ feeling of agency. Agency and the threat thereof are an important topic in chronic pain in general, but also in EM-related CPP [5]. Furthermore, in a chronic pain context, solicitous support may compromise patients’ sense of competence and may even prevent patients from autonomously performing tasks [56]. A review by Rafaeli and Gleason [57] discusses further how the broader relationship context and comparable kinds of social support may threaten the recipients’ feeling of autonomy and thus impact the long-term course of CPP. They emphasize that for support to have a positive impact, it must align with the recipient’s needs and be perceived as satisfactory by them.

Importantly, it should be noted that the relationship between social support and pain might also operate in the opposite direction, where higher pain leads to increased seeking and supportive behaviors. However, based on the outlined literature, the interpretation that specific types of support contribute to higher pain appears more compelling.

Previous research has examined which individual characteristics interact with social support. For example, a meta-analysis found extraversion as the only Big Five personality trait to be significantly associated with received social support [58]. More recently, solicitous support was found to decrease pain in men but not in women [59]. These findings highlight the importance of further investigating how individual differences shape the effects of social support—a crucial step toward developing tailored interventions.

### Cortisol

Contrary to hypothesis 4, self-reported pain levels were not related to salivary cortisol levels. This is consistent with a review on the Trier Social Stress Test, which reported that only 25% of studies found a significant correlation between cortisol response and self-reported stress [60]. One important mediating factor in this inconclusive data might be found in sex hormone variability: Studies have found cortisol responses to fluctuate across the menstrual cycle, with cortisol reactivity being weaker in phases with low estrogen (like in this study; [22]). Furthermore, in the early follicular phase (low estrogen), individuals reported increased psychosocial distress in comparison to the periovulatory phase (high estrogen) [61]. This aligns with our findings of high self-reported (psychosocial) stress alongside low cortisol levels.

### Oxytocin

Higher oxytocin levels were expected to be associated with lower pain levels. However, no significant moment-to-moment link emerged. Most studies in this field predominantly involve healthy male participants, utilize externally administered oxytocin, and employ experimentally induced pain stimuli [41, 42, 62]. Focusing on studies that are more comparable to ours, the findings concerning the relation between endogenous oxytocin and spontaneous pain are mixed. Schneider et al. [63] found oxytocin to be significantly negatively related to emotional pain in women and men. For physical pain, they found a non-significant trend for a negative association, matching findings by Anderberg and Uvnäs-Moberg [64]. Research suggests that women’s oxytocin levels fluctuate across the day [65] and while in our study oxytocin was measured only in the evenings, Schneider et al. [63] took several measures per day enabling them to analyze diurnal variations of the oxytocin-pain relation. Further research is needed to determine if spontaneous physical pain and endogenous oxytocin merely are not related, or if more in-depth studies of diurnal variation are necessary.

### Strengths and Limitations

The design of this study has considerable strengths. The EMA method allowed an assessment of pain processes in a repeated fashion and with great detail, resulting in data of high ecological validity and reliability. Assessing spontaneous real-life pain in relation to daily social interactions and endogenous salivary cortisol and oxytocin during pre-scheduled menstrual cycle phases of high pain variability also contributes to the high ecological validity.

However, the following limitations must be considered: In this study, only distracting and solicitous support were considered. However, other social support styles may also play important roles in the context of chronic pain, as well as the broader relationship context. While EMA approaches bring many advantages, they rely on real-time self-report data that can be susceptible to mood fluctuations and situational influences. Moreover, participants may change their behavior or responses simply due to feeling monitored. Although individuals were pre-screened for high pain levels, their everyday pain ratings were surprisingly low. This discrepancy may be attributed to the strong wording of the label at the scale maximum (“the most extreme pain I have ever experienced”). Potentially, this caused a bias toward lower pain ratings. The number of measurements of oxytocin was limited (396 observations). These measures were taken at the last measure per day with low variance in the data. Likewise, the number of moments, when actual social support was reported, was limited (328 observations). Interpreting the (missing) link between each measure and pain thus requires caution and confirmation through larger sample sizes and more measurement points.

## Conclusion and Implications for Future Research

Distracting as well as solicitous social support were found to be related to higher pain ratings on a between-person level, suggesting a long-term effect within the dyad. More research on different social support styles and their impact is due, especially in CPP samples, where intimate relationships are particularly burdened. Also, there is a need for detailed analyses of the long-term effects of social support styles on recurrent or chronic pain syndromes and how these might be used for pain management and psychosocial interventions. The present data suggests that even well-intended affectionate support for chronic pain patients can actually lead to a worsening of symptoms. In contrast, the slightly positive effects of distraction indicate that couples should be trained to more deliberately incorporate distraction and interactions into their daily routines that are incompatible with pain.

In summary, this study expands previous research suggesting that both stress and social support can have an adverse impact on pain experience in EM. This knowledge is crucial when designing comprehensive interventions for EM patients and including psychosocial aspects: While stress management may be helpful in improving pain symptoms, this is not unconditionally true for social support. Rather, when translating psychosocial interventions to use in patients’ daily lives, particularly close or romantic others need more specific advice on how to respond to pain expressions. Here, some guidance on how to exchange immediate and intuitive supportive behavior with positive feedback on agentic behavior might be beneficial.

## Supplementary Information

Below is the link to the electronic supplementary material.

ESM1 (PDF 112 KB)

ESM2 (PNG 1.18 MB)

ESM3 (PNG 1.09 MB)

ESM4 (PNG 960 KB)

ESM5 (PNG 884 KB)

## Data Availability

To protect participant privacy, the data presented in this study are available from the corresponding author only on request.

## Footnotes

1 https://osf.io/a4etx/files/j7ahq.

2 Endometriosis can affect women as well as non-binary, trans, and intersex individuals. The recruitment materials and our pre-registration were phrased in a gender-neutral way to ensure maximum inclusivity. However, only women participated.

3 https://drks.de/search/de/trial/DRKS00021017.

4 If they did not live together, it was discussed and ensured during the pre-screening that couples met at a regular basis.
