# Supplementary material for: Psychoneuroendocrine Associations with Momentary Pelvic Pain in Endometriosis
Source: Int J Behav Med. 2025 Nov 4;33(2):236–50. doi: 10.1007/s12529-025-10402-w (PMC13161250; doi:10.1007/s12529-025-10402-w)
Supplement: Supplementary file 1 — (PDF 112 KB) [file 12529_2025_10402_MOESM1_ESM.pdf]

EMA-schedule for one day

prompt	time	items used				
P1	upon awakening	saliva sample (cortisol)	control questions + sleep quality			
P2	T1 + 30min	saliva sample (cortisol)	control questions			
P3	T1 + 45min	saliva sample (cortisol)	control questions	mood, social situation, stress, pain		
P4	11:00	saliva sample (cortisol)	control questions	mood, social situation, stress, pain		
P5	14:00	saliva sample (cortisol)	control questions	mood, social situation, stress, pain		
P6	18:00	saliva sample (cortisol)	control questions	mood, social situation, stress, pain		
P7	when going to sleep	saliva sample (cortisol + oxytocin+ Progesterone + estradiol)	control questions	mood, social situation, stress, pain	Catastrophizing, fear of pain, anxiety, activity, coping, partner worries	sexual health (only on day 4+7)

→ on 7 consecutive days

Fig. S1 Schedule of full Ecological Momentary Assessment (EMA). Please note that in the presented study, data from P1 and P2 were not used, so that T1-T5 correspond to P3-P7.
