# Supplementary figures and images for: Psychoneuroendocrine Associations with Momentary Pelvic Pain in Endometriosis
Source: Int J Behav Med. 2025 Nov 4;33(2):236–50. doi: 10.1007/s12529-025-10402-w (PMC13161250; doi:10.1007/s12529-025-10402-w)

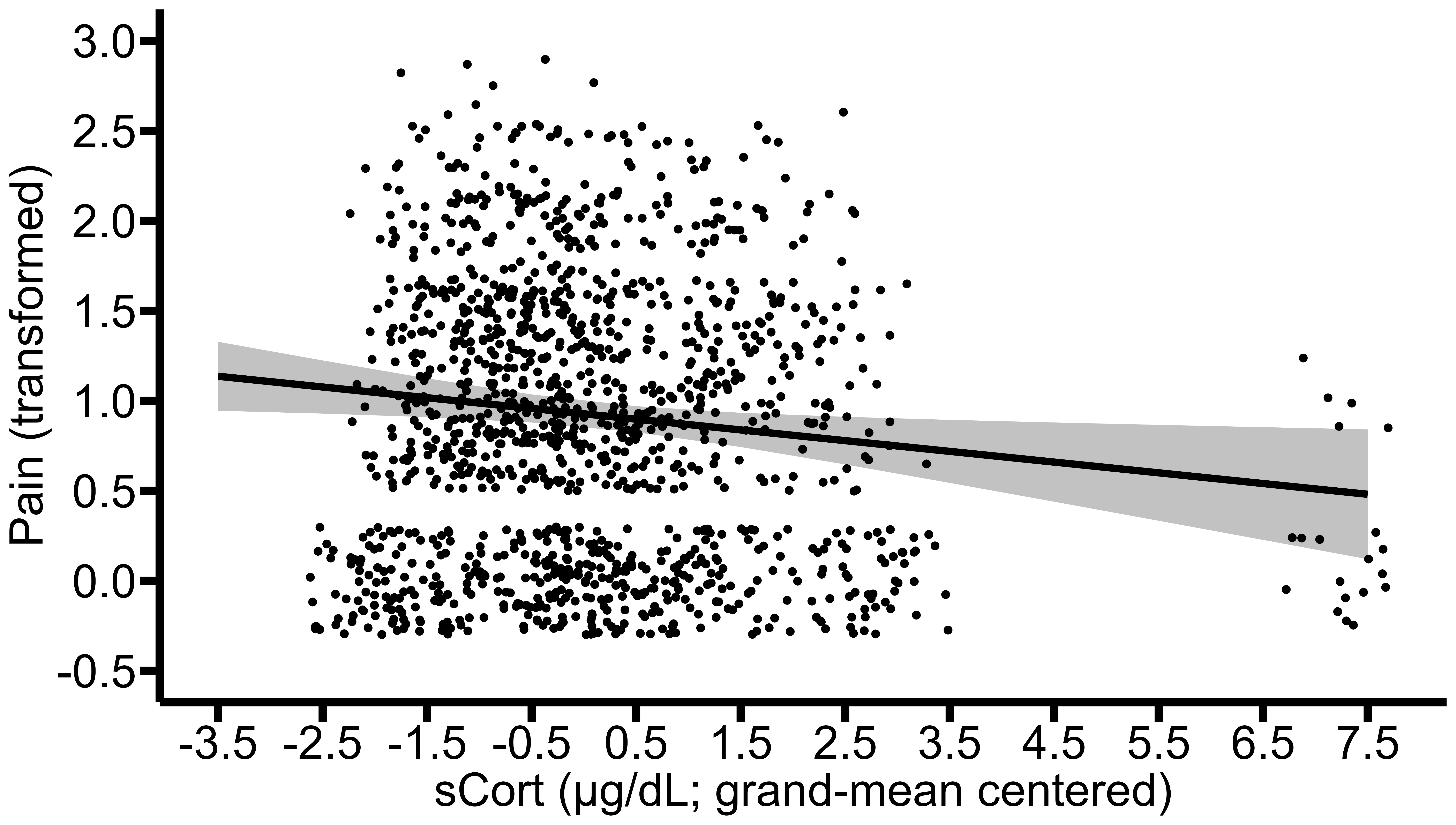

Supplement: Supplementary file 2 — (PNG 1.18 MB) [file 12529_2025_10402_MOESM2_ESM.png]

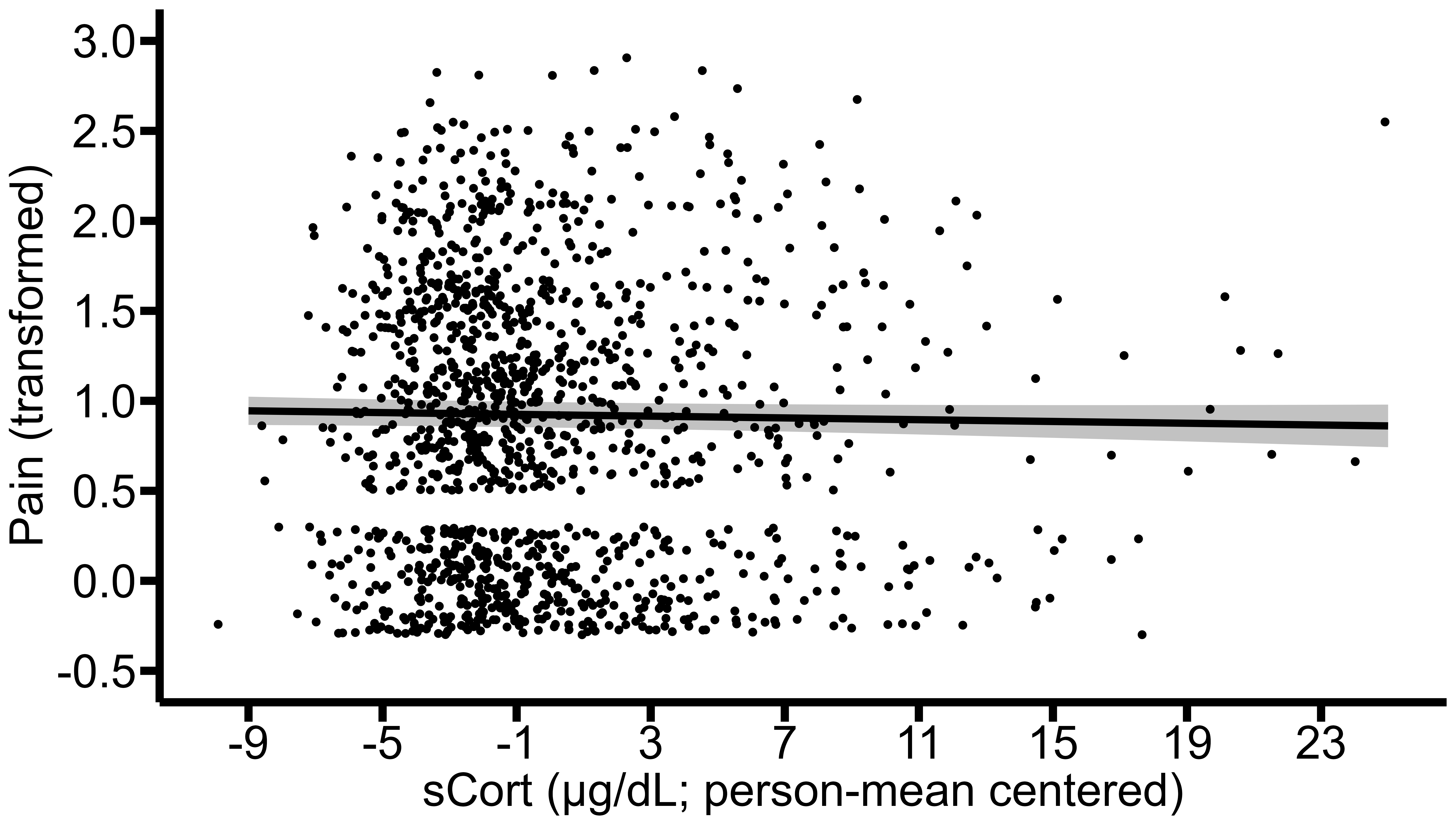

Supplement: Supplementary file 3 — (PNG 1.09 MB) [file 12529_2025_10402_MOESM3_ESM.png]

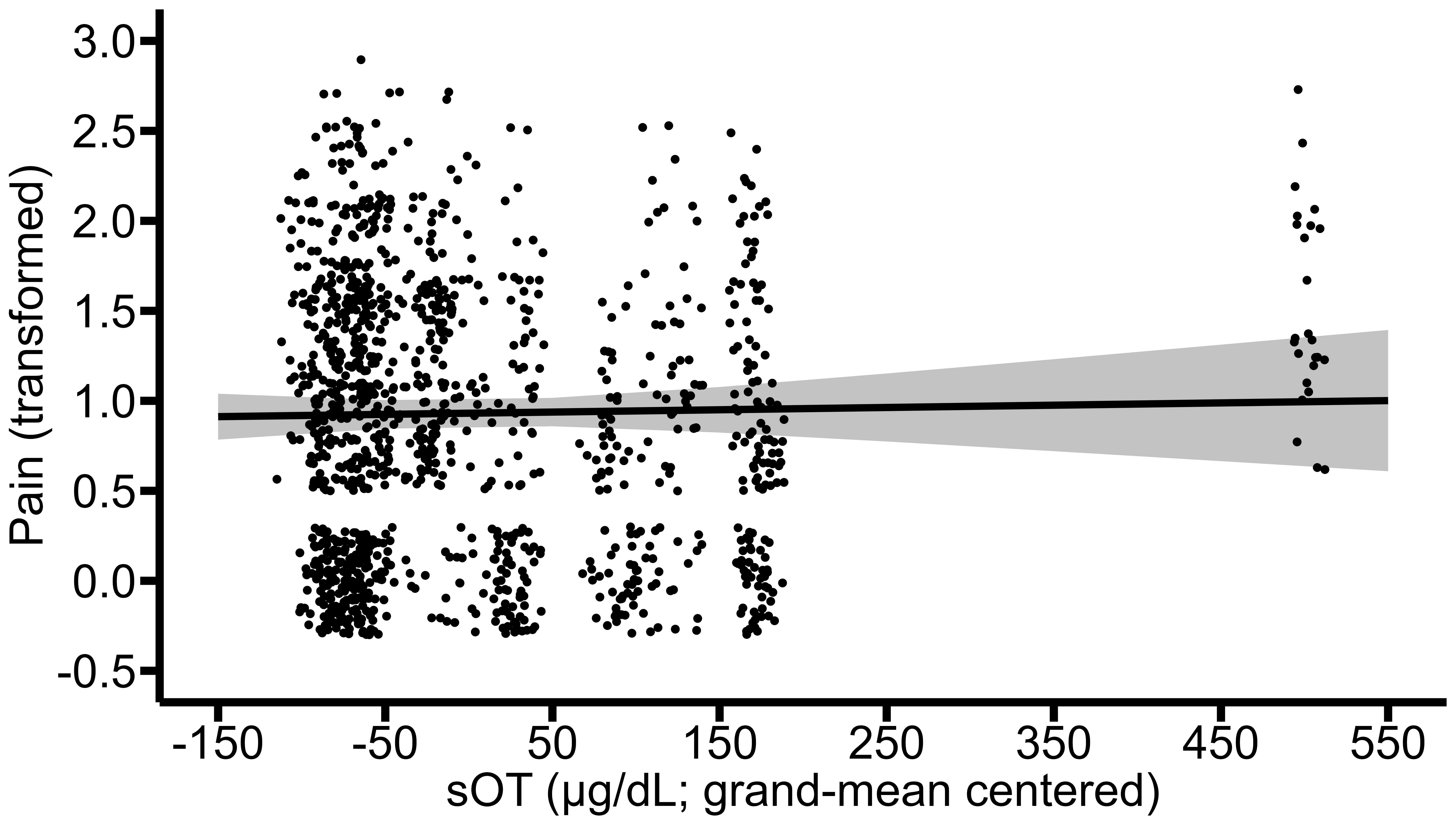

Supplement: Supplementary file 4 — (PNG 960 KB) [file 12529_2025_10402_MOESM4_ESM.png]

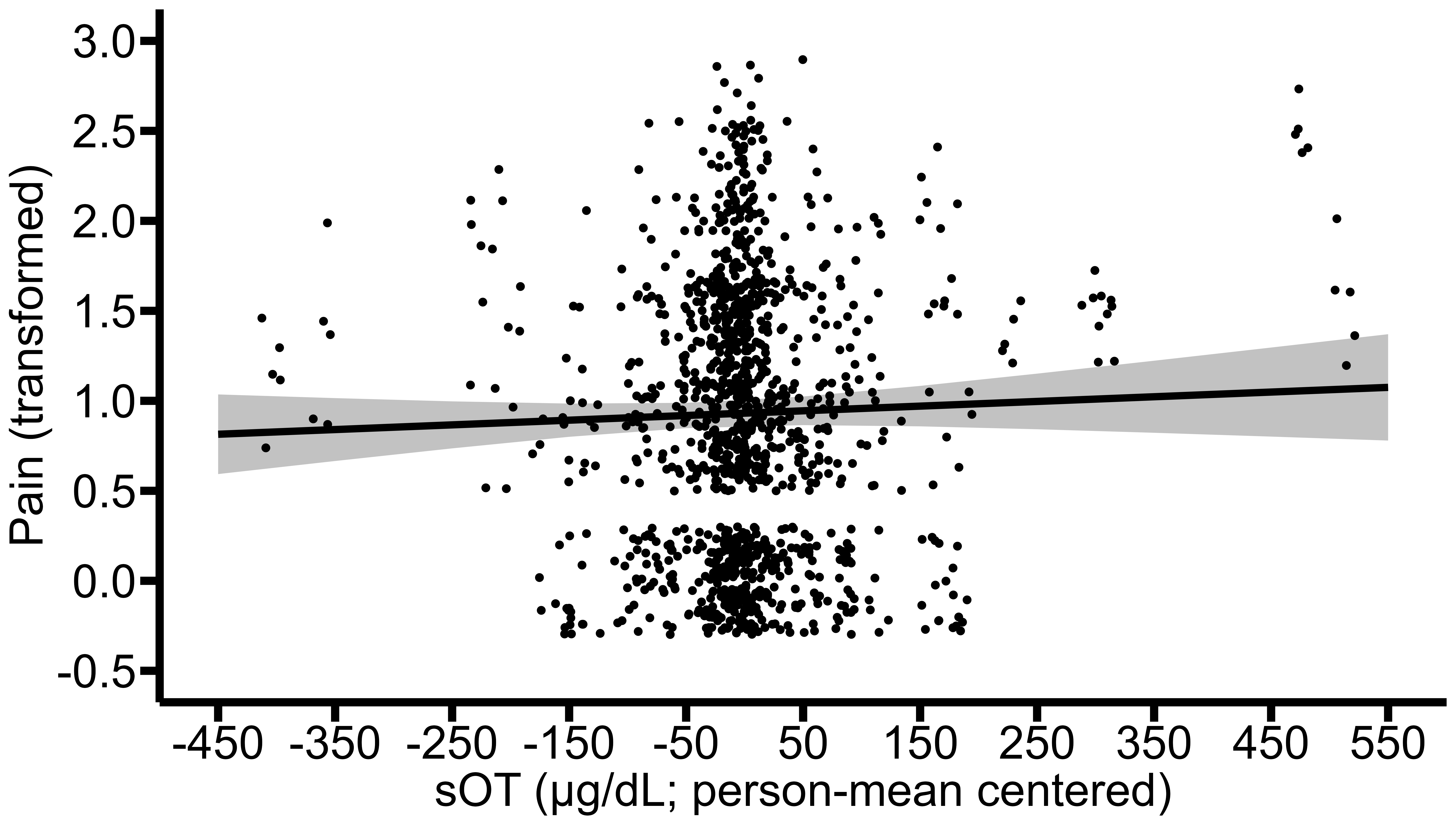

Supplement: Supplementary file 5 — (PNG 884 KB) [file 12529_2025_10402_MOESM5_ESM.png]
